# Impact of artificial intelligence and work digitalization on mental health and occupational well-being: a scoping review

**DOI:** 10.3389/fpubh.2026.1857108

**Published:** 2026-06-01

**Authors:** Óscar Rabasa-Martín, Begoña Martínez-Jarreta

**Affiliations:** 1 Faculty of Medicine, University of Zaragoza, Zaragoza, Spain; 2 Research Group GIIS063-Aragón Health Research Institute (IIS-Aragón), Zaragoza, Spain

**Keywords:** artificial intelligence, digitalization, mental health, occupational health, psychosocial risk, technostress, workplace well-being

## Abstract

**Background:**

The rapid expansion of artificial intelligence (AI) and work digitalization is transforming occupational environments, introducing new psychosocial risks while also creating potential opportunities for improving workplace well-being. However, current evidence remains fragmented and heterogeneous.

**Objective:**

This scoping review aimed to map and synthesize the existing scientific and grey literature on the impact of AI and work digitalization on mental health, well-being, and psychosocial risks among adult workers.

**Methods:**

A scoping review was conducted following the Arksey and O’Malley framework and reported according to PRISMA-ScR guidelines. A comprehensive search was performed across multiple databases (PubMed, Scopus, Web of Science, ScienceDirect, Scielo, LILACS, Dialnet, and Google Scholar) and grey literature sources from international occupational health organizations. Studies published between 2016 and 2026 in English and Spanish were included. A total of 43 sources (23 scientific articles and 20 grey literature documents) were analyzed using thematic synthesis. The review explicitly distinguishes between AI-specific occupational exposures and broader digitalization processes to improve conceptual clarity.

**Results:**

AI and digitalization were consistently associated with multiple psychosocial risks, including technostress, work intensification, job insecurity, reduced autonomy, and blurred work–life boundaries. Algorithmic management and digital monitoring emerged as key drivers of stress, anxiety, and burnout. However, potential benefits were also identified, such as increased efficiency, flexibility, and professional development, particularly when supported by adequate training and organizational resources. The impact of digitalization was context-dependent and unevenly distributed, disproportionately affecting older workers, lower-skilled employees, and vulnerable groups. Digital and AI literacy emerged as key protective factors.

**Conclusion:**

AI and work digitalization represent complex and context-dependent determinants of occupational mental health, with both risks and opportunities depending on organizational, technological, and individual factors. These findings highlight the need for human-centered implementation strategies, strengthened regulatory frameworks, and targeted preventive interventions to mitigate psychosocial risks in digitalized work environments. Given the heterogeneity of the available evidence, findings should be interpreted as exploratory.

## 1 Introduction

The world of work is undergoing profound transformations driven by demographic changes, economic instability, the impact of the COVID-19 pandemic, and the rapid digitalization of work processes. In particular, the integration of artificial intelligence (AI), automation, and digital technologies has emerged as a key determinant of occupational health in the 21st century (1).

Within the European context, Spain can be considered an illustrative example of broader structural challenges affecting occupational health, including labor market instability, adverse working conditions, and an aging workforce. These factors are not unique to Spain but reflect wider trends across European labor markets that may influence the impact of digitalization on workers’ mental health and well-being. This contextual reference is intended solely to illustrate broader structural patterns and does not imply a country-specific focus of the review (2).

Psychosocial factors at work—encompassing organizational structures, job design, management practices, and interpersonal relationships—play a critical role in workers’ health and well-being. Exposure to adverse psychosocial conditions has been linked to increased risks of mental and physical health disorders, absenteeism, reduced productivity, and human error (1–3).

In this context, digitalization has significantly reshaped work environments, altering job roles, working conditions, and occupational risk profiles. In occupational health practice, these technological advances offer new opportunities for risk prevention, health surveillance, and the optimization of clinical and preventive processes (4). However, they also introduce emerging challenges, including work intensification, ergonomic and musculoskeletal risks associated with automation, cybersecurity threats, and psychosocial stressors (5, 6), as well as additional physical and ergonomic risks in highly automated environments (7, 8).

Artificial intelligence, in particular, plays an ambivalent role in occupational health. On the one hand, it enables more proactive, data-driven approaches to prevention and decision-making. On the other, its implementation may generate tensions related to professional autonomy, decision-making processes, and organizational control, especially when integrated into complex work systems (9–11).

The integration of AI into health-related fields further illustrates its potential to transform decision-making processes, while also raising concerns regarding reliability, safety, and professional responsibility (12–15).

From an occupational health perspective, artificial intelligence (AI) can be understood as a set of data-driven systems capable of performing tasks that typically require human cognitive functions, including decision-making, prediction, and pattern recognition. In workplace contexts, AI is particularly relevant as a mechanism of algorithmic decision-making, monitoring, and task allocation, which directly shapes psychosocial exposures and working conditions (16, 17).

The rapid expansion of digital technologies within the context of the Fourth Industrial Revolution has profoundly transformed the organization and nature of work. These transformations have significant implications for mental health, as changes in job design, work intensity, and organizational control mechanisms directly influence psychosocial risk exposure. Emerging evidence suggests that digitalized work environments may contribute to increased stress, burnout, and work–life imbalance, particularly in contexts characterized by high demands and reduced autonomy (18–21).

The literature has identified several key psychosocial risks associated with digitalized work environments, including job insecurity, technostress, social isolation, role ambiguity, work–life boundary blurring, and algorithmic surveillance. These factors have been consistently associated with adverse mental health outcomes, such as anxiety, burnout, sleep disturbances, and reduced overall well-being (20, 22–28).

While digitalization offers substantial benefits, its impact on occupational health is highly dependent on organizational, regulatory, and individual factors. Evidence suggests that the negative effects of digital work can be mitigated through appropriate management strategies, digital competencies, and preventive interventions aimed at reducing digital stress and enhancing worker autonomy (29).

In parallel, the growing implementation of AI in occupational settings raises important ethical and regulatory challenges. Concerns related to algorithmic transparency, data protection, bias, and professional responsibility have led to the development of regulatory frameworks at both European and national levels, including the European Union Artificial Intelligence Act (30–35), which aims to ensure the safe, ethical, and human-centered use of AI technologies.

Although artificial intelligence and broader processes of work digitalization are conceptually distinct, they are closely interconnected in contemporary occupational settings. Digitalization encompasses a wide range of technologies that transform work organization, including remote work systems, platform-based labor, and electronic monitoring, while AI represents a more specific subset of these technologies characterized by data-driven decision-making and automation capabilities (16). In practice, these processes often co-occur and interact, jointly shaping working conditions, job design, and psychosocial risk exposure. For this reason, this review adopts an integrated analytical approach, while maintaining a conceptual distinction between AI-specific mechanisms and broader digitalization processes. In this review, this distinction was operationalized during data extraction and synthesis by classifying exposures as AI-specific or broader digitalization-related where possible.

Despite the growing body of literature on artificial intelligence and work digitalization, important conceptual and empirical gaps remain. In particular, existing studies often fail to clearly distinguish between AI-specific exposures—such as algorithmic management, automation-related job insecurity, and AI-driven decision-making—and broader processes of digitalization, including the use of information and communication technologies, telework, and platform-based work. Moreover, the current evidence base is highly fragmented across disciplines, methodologies, and types of sources, with limited integration between scientific literature and institutional reports. This fragmentation hinders a comprehensive understanding of how these technologies influence mental health and psychosocial risks in occupational settings. To our knowledge, no previous scoping review has systematically integrated both AI-specific and broader digitalization-related exposures within a unified analytical framework while explicitly addressing their differential impact on psychosocial risks and mental health outcomes.

Therefore, this scoping review seeks to address these gaps by providing an integrated overview of the main psychosocial risks, contextual factors, and potential opportunities associated with AI and work digitalization.

## 2 Objective

This scoping review aims to systematically map and synthesize the existing scientific literature on the impact of artificial intelligence and work digitalization on mental health, occupational well-being, and psychosocial risks among adult workers. It also aims to identify key psychosocial risks and potential opportunities, examine the organizational contexts in which these technologies are implemented, and highlight research gaps to inform future research and occupational health practice.

## 3 Methods

### 3.1 Study design

A scoping review was conducted following the methodological framework proposed by Arksey and O’Malley and further refined by Levac et al. and Daudt et al. (36–41). The review was reported in accordance with the Preferred Reporting Items for Systematic Reviews and Meta-Analyses extension for Scoping Reviews (PRISMA-ScR) guidelines (36).

### 3.2 Research question

The review was guided by the Population–Concept–Context (PCC) framework recommended by the Joanna Briggs Institute. The main research question was:

How has the impact of artificial intelligence and work digitalization on mental health, well-being, and psychosocial risks been addressed in the scientific literature?

Secondary questions explored psychosocial risks and opportunities, occupational contexts, generational factors, and the role of digital and AI literacy.

The PCC framework used to define the scope of the review is presented in Table 1.

**Table 1:** PCC framework.

Component	Description
Population	Adult workers (≥18 years) in any occupational sector
Concept	Impact of AI and digitalization on mental health, well-being, and psychosocial risks
Context	Digitalized workplaces and environments using AI-based systems

### 3.3 Search strategy

A comprehensive literature search was conducted across the following electronic databases: PubMed, Scopus, Web of Science, ScienceDirect, Scielo, LILACS, Dialnet, and Google Scholar.

The search was conducted between January 2016 and January 2026, with the final search performed on 15 January 2026.

Search terms were developed using a combination of controlled vocabulary (e.g., Medical Subject Headings [MeSH]) and free-text keywords related to artificial intelligence, digitalization, occupational health, and mental health. Boolean operators (AND, OR) were used to combine search terms.

The search strategy was as follows:

(“Artificial Intelligence”[MeSH] OR “AI” OR “machine learning” OR “algorithm” OR “automation”) AND (“Digitization” OR “Digital Transformation” OR “Industry 4.0” OR “digital technology”) AND (“Occupational Health”[MeSH] OR “Workplace” OR “Work Environment” OR “occupational safety”) AND (“Mental Health”[MeSH] OR “psychosocial factors” OR “psychological stress” OR “well-being” OR “burnout”)

This strategy was adapted for each database by adjusting keywords and controlled vocabulary as appropriate. Minor variations in syntax were applied depending on database-specific requirements, while maintaining consistency in the core concepts of artificial intelligence, digitalization, occupational health, and mental health.

The number of records retrieved from each database is presented in Table 2.

**Table 2:** Databases and number of records retrieved.

Database	Records retrieved
Dialnet Plus	49
Google Scholar	88
LILACS	85
PubMed	142
Scielo	35
ScienceDirect	83
Scopus	68
Web of Science	48

Grey literature searches were conducted between January 2016 and January 2026 through systematic and targeted searches of official websites of relevant national and international organizations in the field of occupational health and digitalization. Predefined keywords were used alongside manual screening of institutional repositories. Documents were selected based on predefined relevance criteria aligned with the objectives of the review, focusing on reports, policy documents, and technical publications addressing artificial intelligence, digitalization, and psychosocial risks in work environments.

The number of records retrieved from these sources is presented in Table 3.

**Table 3:** Grey literature sources and records retrieved.

Source	Records retrieved
European Agency for Safety and Health at Work (EU-OSHA)	9
European Foundation for the Improvement of Living and Working Conditions (EUROFOUND)	5
European Trade Union Institute (ETUI)	3
Eurostat (86)	1
Finnish Institute of Occupational Health (FIOH) (87)	1
Health and Safety Executive (HSE) (88)	3
Institution of Occupational Safety and Health (IOSH) (89)	5
Instituto Nacional de Seguridad y Salud en el Trabajo (INSST)	4
Joint Research Centre (JRC)	3
National Institute for Occupational Safety and Health (NIOSH) (90)	2
Organization for Economic Co-operation and Development (OECD) (91)	5
International Labour Organization (ILO) (85)	3
World Health Organization (WHO) (92)	2
United Nations Economic Commission for Europe (UNECE)	1

The full search strategies for each database, including database-specific adaptations of keywords, controlled vocabulary, and syntax, are provided in Supplementary material (Appendix 1) to ensure transparency and facilitate reproducibility.

### 3.4 Eligibility criteria

Study inclusion and exclusion criteria were defined *a priori* to ensure consistency and transparency in the selection process.

No restrictions were applied regarding study design, population, or geographical setting in order to capture the breadth of available evidence. Language was limited to English and Spanish.

The inclusion and exclusion criteria are summarized in Table 4.

**Table 4:** Inclusion and exclusion criteria.

Inclusion criteria	Exclusion criteria
Adult workers (≥18 years) in digitalized or AI-based work environments Studies addressing mental health, well-being, or psychosocial risks Any study design (quantitative, qualitative, mixed methods) Published between 2016 and 2026 English or Spanish	AI or digitalization not central to the study No occupational or psychosocial outcomes Non-work-related settings Non-analytical or purely opinion-based documents

Given the exploratory nature of this scoping review, a wide range of evidence types was included, encompassing primary studies, systematic and narrative reviews, methodological studies, and institutional reports. This inclusive approach is consistent with scoping review methodology and allows for a comprehensive mapping of both empirical scientific evidence and policy-oriented or institutional perspectives, which is particularly relevant in emerging fields where grey literature plays a complementary role. To address potential overlap between primary studies and secondary reviews, each source was analyzed independently based on its contribution to the research questions, focusing on identifying key themes and patterns rather than quantifying individual study findings. No attempt was made to quantitatively aggregate findings across studies, and potential overlap between primary studies and secondary reviews was considered an inherent limitation of the mapping approach.

### 3.5 Study selection

All records were imported into the Mendeley reference manager for organization and duplicate removal.

The study selection process was conducted in three stages: (1) removal of duplicates, (2) screening of titles and abstracts based on predefined eligibility criteria, and (3) full-text review to confirm inclusion.

Titles and abstracts were screened independently by two reviewers according to the predefined eligibility criteria. Full-text articles were then assessed for eligibility. Any disagreements between reviewers were resolved through discussion until consensus was reached. This process ensured a systematic, transparent, and reproducible identification of relevant studies. The selection process is presented using a PRISMA flow diagram.

### 3.6 Data extraction

Data were extracted using a standardized Excel template to ensure consistency across studies. The following variables were collected: author(s), year, and source; country or region; study design or type of document; sample size or number of included sources; key variables; type of AI or digitalization (when applicable); outcomes related to mental health and psychosocial risks; and main findings.

Data extraction was performed independently by two reviewers using the standardized template. Discrepancies were discussed and resolved by consensus to ensure accuracy and consistency of the extracted information.

### 3.7 Data synthesis

A descriptive and narrative synthesis was conducted to systematically map the available evidence. Findings were grouped into thematic categories, including psychosocial risks, occupational contexts, preventive strategies, positive effects and opportunities, inequalities and vulnerable groups, and emerging research gaps.

A thematic synthesis approach was applied to identify and organize key patterns across the included studies. This process followed an inductive approach, allowing themes to emerge from the data rather than being predefined.

Where relevant, findings were interpreted by distinguishing between empirical scientific studies and institutional or grey literature sources, allowing for a more nuanced understanding of the evidence base.

In line with established methodological guidance for scoping reviews, a formal quality assessment of included sources was not conducted. The primary objective of this study was to map the breadth, nature, and characteristics of the available evidence rather than to evaluate the methodological quality or strength of specific findings. Furthermore, the included evidence was highly heterogeneous, encompassing empirical studies with different designs, reviews, and institutional reports, which limits the applicability and comparability of standardized quality appraisal tools. Conducting separate quality assessments for each type of evidence was considered beyond the scope of this review and potentially inconsistent with its exploratory purpose. Nevertheless, the absence of formal appraisal may introduce variability in the robustness of the evidence, and therefore the findings should be interpreted as descriptive and exploratory rather than conclusive.

## 4 Results

### 4.1 Study selection

The final search yielded 645 records, including 598 from scientific databases and 47 from grey literature sources.

After removing 213 duplicates, 385 records remained for screening. Following title and abstract screening, 197 records were excluded, resulting in 188 full-text articles assessed for eligibility.

Of these, 165 were excluded for not meeting the inclusion criteria, leaving 23 studies from the scientific literature.

For grey literature, 47 records were identified and assessed, of which 27 were excluded, resulting in 20 included documents.

In total, 43 studies were included in this scoping review, comprising 23 scientific articles and 20 grey literature sources.

**Figure 1:**
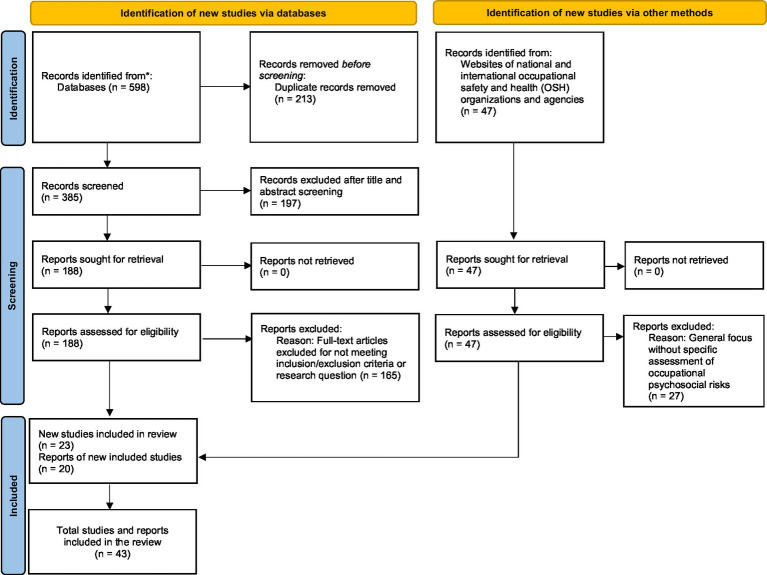
PRISMA-ScR flow diagram illustrating the study selection process for scientific and grey literature sources.

The study selection process is illustrated in the PRISMA-ScR flow diagram (Figure 1).

### 4.2 Characteristics of included studies

The included studies showed considerable heterogeneity in terms of study design, geographical distribution, and occupational contexts. Most studies were observational and conducted in high-income countries, with a strong representation of European and Asian contexts. The evidence covered a wide range of sectors, including healthcare, administrative work, platform-based employment, and industrial settings.

The characteristics of the included studies are summarized in Tables 5, 6. Table 5 presents the main features of the scientific literature (*n* = 23), while Table 6 summarizes the grey literature sources (*n* = 20), including institutional reports and policy documents.

**Table 5:** Characteristics of included scientific studies on artificial intelligence and work digitalization (*n* = 23).

Author (Year)	Country	Study design	Sample	Key variables	Main findings
Artificial intelligence (AI)					
**Empirical studies**					
Gao and Zamanpour (23)	China/Iran	Mixed-methods study	321 workers	Psychological safety, work–life balance	AI may improve efficiency but can increase stress depending on role
Pizzi and Payá Castiblanque (43)	Europe	Cross-sectional study	27,252 workers	AI monitoring, stress, autonomy	AI surveillance associated with higher stress and lower autonomy
Jin et al. (25)	China	Cross-sectional study	349 workers	STARA awareness, stress	Perceived AI threat associated with reduced well-being via stress
Yoon et al. (75)	Singapore	Mixed-methods study	399 participants	Well-being, usability	AI platforms may support well-being when properly designed
Giuntella et al. (42)	Germany	Longitudinal study	Panel data	AI exposure, health	No significant negative effects on mental health observed
Zhang et al. (66)	China	Quantitative study	381 workers	Technostress, innovation	Technostress may hinder or enhance performance depending on perception
Nofal et al. (45)	Middle East	Quasi-experimental study	90 workers	Training, well-being	AI training associated with improved well-being and professional development
Wei & Li (24)	China	Cross-sectional study	Not specified	Mental health, overtime	AI associated with poorer mental health via increased workload
Niehaus et al. (44)	Germany	Cross-sectional study	6,153 workers	Control, workload, burnout	Algorithmic control reduces autonomy and increases burnout
**Review studies**					
Cramarenco et al. (46)	International	Systematic review	Not applicable	Well-being, skills	AI shows both positive and negative effects depending on context
Fisher et al. (47)	International	Scoping review	Not applicable	Equity, risks	AI may exacerbate occupational health inequalities
Digitalization and work environments					
**Empirical studies**					
Ficapal-Cusí et al. (22)	Spain	Cross-sectional study	311 healthcare workers	Technostress, burnout	Digitalization associated with increased technostress and burnout
Holmgren et al. (56)	USA	Cross-sectional study	~6,000 physicians	EHR usability, burnout	Poor usability associated with higher burnout
Parteka et al. (58)	Europe	Quantitative study	Large datasets	Work intensity	Digitalization associated with increased work intensification
Arana-Landín et al. (59)	Europe	Quantitative study	Not specified	Safety, workload	Improves safety but increases workload demands
Borle et al. (57)	Germany	Cross-sectional study	Not specified	ICT exposure, health	Work intensification linked to poorer health outcomes
**Review and methodological studies**					
Kim and Cho (7)	South Korea	Narrative review	Not applicable	Physical and psychosocial risks	Digitalization linked to both physical and psychosocial risks
Schlenger et al. (64)	Germany	Instrument validation study	Not applicable	Digital stressors	Valid tool developed for assessing digital psychosocial risks
Rodríguez (65)	International	Comparative study	Not applicable	Risk assessment tools	Existing tools insufficient for digital work environments
Håkansta et al. (76)	International	Scoping review	Not applicable	Occupational health	Mixed effects: efficiency gains vs. increased stress
Dragano & Lunau (60)	International	Narrative review	Not applicable	Technostress	Strong association between technostress and mental health
Teodoroski et al. (8)	Brazil	Narrative review	Not applicable	Automation, risks	Automation reduces physical risks but introduces psychosocial risks
Komp-Leukkunen et al. (77)	International	Systematic review	Not applicable	Aging, work	Digitalization creates both risks and opportunities for older workers

AI, artificial intelligence; ICT, information and communication technologies; EHR, electronic health records. Studies are grouped by type (empirical vs review/methodological) to improve comparability and interpretability of findings.

**Table 6:** Characteristics of included grey literature sources on artificial intelligence and work digitalization (*n* = 20).

Organization	Included resources (n)	Type of documents	Years / Region	Focus	Key psychosocial findings
European Agency for Safety and Health at Work (EU-OSHA)	6	Reports, policy briefs, technical documents	2021–2025 (EU)	Digitalization, AI, OSH	Identifies technostress, workload, reduced autonomy, isolation, and work intensification as key risks; emphasizes the need for preventive policies
International Labour Organization (ILO)	3	Reports and policy documents	2022–2025 (Global)	Mental health at work, digitalization	Identifies AI and digitalization as emerging determinants of psychosocial risks; promotes global prevention frameworks
Instituto Nacional de Seguridad y Salud en el Trabajo (INSST)	3	Technical reports and guidelines	2022–2024 (Spain)	Telework, platform work	Highlights isolation, workload, technostress, job insecurity, and algorithmic control; proposes preventive strategies
European Foundation for the Improvement of Living and Working Conditions (Eurofound)	3	Analytical reports (survey-based)	2020–2023 (EU)	Telework, platform economy	Identifies associations with stress, work–life imbalance, isolation, and increased mental load
Joint Research Centre (JRC)	3	Technical and analytical reports	2021–2024 (EU)	Algorithmic management	Identifies surveillance, reduced autonomy, and increased job demands as key psychosocial risks
European Trade Union Institute (ETUI)	1	Analytical report	2024–2025 (EU)	AI and worker health	Highlights algorithmic management, workload intensification, and lack of transparency as key risk factors
United Nations Economic Commission for Europe (UNECE)	1	Policy brief	2021 (Europe)	Aging and digitalization	Highlights inequalities, digital divide, and increased vulnerability among older workers

In Table 5, studies are organized by thematic domain (artificial intelligence and work digitalization) and further grouped by type of evidence (empirical and review/methodological studies) to enhance clarity and facilitate comparison across sources.

The majority of included empirical studies were cross-sectional in design, with a smaller proportion of mixed-methods and quantitative studies, and only a limited number of longitudinal or quasi-experimental designs. This distribution reflects the emerging nature of research in this field and highlights the predominance of observational approaches over longitudinal or causal analyses.

### 4.3 AI and perceived threat

Empirical studies consistently identify perceived technological threat as a central mechanism underlying psychosocial risk. Awareness of automation and AI-related job substitution is consistently associated with increased stress and reduced well-being, often mediated by perceived job insecurity and workload (23–25, 42–47), particularly in studies such as Jin et al. (25) and Gao and Zamanpour (23).

Institutional evidence, particularly from the European Agency for Safety and Health at Work (EU-OSHA) and the Joint Research Centre (JRC), reinforces these findings, highlighting job insecurity, reduced autonomy, and increased performance monitoring as key risk factors in AI-driven work environments (48–51).

Algorithmic management is consistently associated across both empirical studies and institutional reports with reduced decision-making capacity, increased time pressure, and intensified job demands, contributing to stress, anxiety, and depressive symptoms (49, 52–55), as also emphasized by the European Trade Union Institute (ETUI) (55).

These findings were predominantly supported by empirical studies, while institutional reports emphasized broader structural and organizational dimensions of psychosocial risk in AI-driven work environments.

### 4.4 Digitalization, technostress, and work intensification

A substantial proportion of empirical studies indicate that digitalization is associated with work intensification, cognitive overload, and technostress. Digital systems, particularly in healthcare settings, are linked to burnout, reduced job satisfaction, and increased administrative burden when poorly integrated into workflows (22, 56–60), as reported by Ficapal-Cusí et al. (22) and Holmgren et al. (56).

Institutional evidence, particularly from EU-OSHA and the Joint Research Centre (JRC), further confirms that digital transformation modifies work organization, increasing time pressure, workload, and cognitive demands, while contributing to fatigue, digital overload, and work–life imbalance (48, 49, 51, 61, 62).

Large-scale European data from EU-OSHA indicate a high prevalence of psychosocial risks, including time pressure (44%), lack of rewards (34%), and poor organizational communication (29%), suggesting widespread exposure to psychosocial stressors in digitalized work environments (62, 63).

Collectively, these findings were consistently supported by both empirical studies and institutional reports, with the latter emphasizing the widespread and structural nature of psychosocial risks associated with digitalized work environments.

### 4.5 Telework and platform-based work

Empirical evidence indicates that telework and platform-based work represent telework and platform-based work as high-risk contexts for psychosocial exposure. Communication challenges, role ambiguity, and insufficient assessment tools have been reported as key limitations in digital work environments (64–66), particularly in studies such as Schlenger et al. (64) and Rodríguez (65).

Institutional evidence, especially from Eurofound and EU-OSHA, further highlights that telework is associated with social isolation and blurred work–life boundaries (67, 68), as well as increased mental load and “virtual presenteeism” (63, 69, 70).

Platform-based work and algorithmic task allocation, as reported in institutional sources such as the Instituto Nacional de Seguridad y Salud en el Trabajo (INSST), are also linked to job insecurity, fragmented work patterns, and intensified performance control, contributing to stress and mental fatigue (71–74).

Taken together, these findings were supported by both empirical studies and institutional reports, with the latter placing greater emphasis on structural conditions such as employment precarity, organizational control, and the regulatory challenges of platform-based work.

### 4.6 Positive effects and opportunities

A growing body of literature highlights that, despite the identified risks, AI and digitalization may also generate important benefits for workers and organizations.

Empirical studies report mixed but often positive outcomes, including no significant negative effects on mental health in some contexts, as well as improvements in efficiency, professional development, and workplace well-being when adequate training and organizational support are provided (42, 45, 46, 75), particularly in studies such as Giuntella et al. (42) and Nofal et al. (45).

Evidence from institutional sources, particularly EU-OSHA and Eurofound, further emphasizes that digital technologies may enhance productivity, flexibility, and access to services, especially when implemented within supportive organizational frameworks (48, 63, 68).

Overall, findings from both empirical studies and institutional reports converge in suggesting that the impact of digitalization is highly context-dependent. While empirical evidence focuses on individual and organizational outcomes, institutional reports place greater emphasis on enabling conditions such as training, governance, and regulatory frameworks.

### 4.7 Inequalities, aging, and digital literacy

Evidence across both scientific and grey literature indicates that the impact of digitalization is unevenly distributed across populations.

Empirical studies highlight greater vulnerability among lower-skilled workers, older employees, and socioeconomically disadvantaged groups (24, 47, 57, 76, 77), as reported by Wei and Li (24) and Borle et al. (57).

Institutional evidence, particularly from EU-OSHA, UNECE, and Eurofound, further reinforces these findings, indicating that digital transformation may exacerbate generational and structural inequalities, especially in the absence of adequate training and organizational support (48, 68, 69, 78, 79).

Digital and AI literacy consistently emerge as key protective factors. Institutional sources, including EU-OSHA, the International Labour Organization (ILO), and UNECE, emphasize that limited understanding of digital systems increases uncertainty, anxiety, and technostress, whereas adequate training enhances perceived control, competence, and well-being (62, 80–83).

These findings from both empirical studies and institutional reports converge in suggesting that digital literacy functions not only as a skill but as a key determinant of occupational health, shaping workers’ capacity to adapt to, interact with, and benefit from digitalized work environments.

## 5 Discussion

This scoping review provides a structured synthesis of the current evidence on the impact of artificial intelligence (AI) and work digitalization on mental health, well-being, and psychosocial risks. The findings highlight a complex and heterogeneous field in which technological transformation interacts with organizational, social, and individual factors, shaping diverse and context-dependent outcomes.

Given the exploratory nature of this scoping review and the heterogeneity of the included evidence, the findings should be interpreted as indicative of emerging patterns rather than definitive conclusions. Accordingly, causal interpretations should be avoided, and findings should be understood as indicative patterns rather than definitive associations.

### 5.1 Principal findings

The evidence suggests that digitalization and AI are associated with multiple psychosocial risks, including technostress, work intensification, reduced autonomy, and job insecurity (20, 22–25, 42–47, 61). Institutional evidence further supports these findings (48–51). However, these effects are not uniform and appear to depend on how technologies are implemented and integrated into work environments.

A key mechanism underlying these effects is the perception of technological threat. Previous studies suggest that awareness of automation may negatively affect well-being through increased stress levels (25), while AI adoption has also been associated with blurred work–life boundaries and increased psychological demands (23).

At the same time, some studies report no significant negative effects on mental health, particularly in longitudinal analyses, as observed by Giuntella et al. (42), further highlighting the heterogeneity of findings and the importance of contextual factors.

From an institutional perspective, reports from organizations such as the European Agency for Safety and Health at Work and the Joint Research Centre highlight job insecurity, increased monitoring, and reduced autonomy as key emerging psychosocial risks in AI-driven workplaces (48–50).

These findings indicate that digitalization should not be conceptualized as a uniform risk factor, but rather as a context-dependent determinant of occupational health. Psychosocial risks do not appear to be inherent to digital technologies per se, but instead emerge from the interaction between technological systems, organizational design, implementation strategies, and working conditions.

### 5.2 Interpretation in light of theoretical models

The findings of this review can be interpreted within established occupational health frameworks, providing a structured approach to understanding how digitalization may influence workers’ mental health.

Consistent with the Job Demand–Control–Support model, the evidence suggests that algorithmic management and digital monitoring systems may increase job demands while simultaneously reducing autonomy and social support (43, 44). This imbalance is likely to contribute to increased psychosocial strain, particularly in highly controlled and performance-driven environments.

Similarly, the Job Demands–Resources model helps explain the ambivalent nature of digitalization observed in the results. While increased demands, such as cognitive overload and constant connectivity, are associated with stress and burnout (60), the availability of resources, including training, organizational support, and digital literacy, may mitigate these effects and potentially promote well-being.

Institutional reports further support this interpretation, indicating that digital transformation may reshape both work organization and psychosocial exposures, with outcomes largely dependent on how technologies are implemented and managed within organizations (55, 81, 84).

These models reinforce the interpretation that the impact of digitalization is not inherently harmful or beneficial, but depends on the balance between demands and resources within specific work contexts.

### 5.3 Heterogeneity and contextual factors

One of the most relevant findings of this review is the considerable heterogeneity of outcomes across studies.

While some evidence suggests negative effects on mental health (24, 25, 43), other studies report neutral or even positive impacts. For example, AI-based training interventions have been associated with improved professional development and well-being (45), and digital tools may support mental health when appropriately designed and implemented (75).

This variability is further supported by institutional evidence, which highlights that digitalization may enhance flexibility and productivity while simultaneously introducing new psychosocial risks, depending on working conditions and organizational context (68, 69).

Part of this heterogeneity may also reflect differences between empirical scientific studies and institutional reports, which often emphasize broader systemic trends and policy-relevant risks.

Several factors may help explain this heterogeneity. Differences in study design, particularly between cross-sectional and longitudinal approaches, may contribute to inconsistencies in findings, as short-term associations may not fully capture longer-term effects. In addition, variability in how digitalization and AI exposure are defined and measured limits comparability across studies, ranging from specific technologies such as algorithmic management to broader processes of digital transformation.

Occupational context also appears to play a critical role. The impact of digitalization may vary across sectors, job types, and levels of task complexity, with high-demand and low-control environments showing greater susceptibility to psychosocial risks.

These findings suggest that the psychosocial impact of AI and digitalization is strongly context-dependent, reflecting the dynamic interaction between job design, autonomy, resource availability, and organizational support structures.

### 5.4 Vulnerable groups and inequalities

The evidence suggests that the impact of digitalization is unevenly distributed across the workforce, disproportionately affecting certain population groups.

Workers who are older, lower-skilled, or socioeconomically disadvantaged appear to be particularly vulnerable to digital stressors, facing greater challenges in adapting to rapidly changing technological environments (24, 57, 77). These vulnerabilities are not solely individual but are closely linked to structural factors, including access to training, job security, and digital resources.

Institutional evidence further supports this perspective, highlighting that digitalization may exacerbate existing inequalities related to age, gender, and access to digital technologies, particularly in the absence of inclusive organizational policies and support mechanisms (48, 62, 78).

These findings indicate that digital transformation may reinforce pre-existing social and occupational inequalities if not accompanied by targeted and inclusive preventive strategies, particularly those aimed at improving access to training, digital competencies, and organizational support.

### 5.5 Implications for occupational health and prevention

From an occupational health perspective, these findings suggest the importance of integrating psychosocial risk management into the design and implementation of digital technologies.

Rather than focusing solely on technological innovation, organizations may benefit from adopting a more holistic approach that considers work design, employee autonomy, and psychosocial working conditions. In particular, the integration of psychosocial risk assessment into AI systems and digital workflows may represent a relevant priority (48–50).

Digital and AI literacy also appear to function as important protective factors. Evidence suggests that adequate training may reduce uncertainty, enhance perceived control, and mitigate technostress, thereby supporting workers’ mental health and well-being (62, 80–83).

In addition, maintaining a human-centered approach appears to be an important consideration. The preservation of professional judgment, human oversight, and ethical considerations in AI implementation may help prevent excessive automation and loss of control in the workplace (5, 16).

These findings support the need to integrate psychosocial risk assessment into the design and implementation of digital systems, rather than addressing such risks solely at later stages.

Recent applied research, including studies published after the search period, suggests an increasing use of AI-driven systems for monitoring worker well-being and predicting burnout risk. While these approaches highlight the potential of digital technologies to support the early detection of psychosocial risks, they also underscore the importance of ensuring ethical use, transparency, and adequate worker protection in their deployment.

### 5.6 Strengths and limitations

This review has several strengths. It provides a structured and comprehensive synthesis of both scientific and grey literature, allowing for a broad and inclusive overview of the topic. The use of a scoping review methodology following PRISMA-ScR guidelines enhances transparency and reproducibility.

However, several limitations should be acknowledged. First, the absence of a formal quality assessment reflects the exploratory nature of the review and the heterogeneity of the evidence base, and may introduce variability in the robustness of the included evidence. Second, the predominance of cross-sectional study designs may limit causal inference (36–38), and the heterogeneity of study designs complicates direct comparisons across studies. Additionally, the inclusion of heterogeneous evidence types may limit direct comparability across sources. Third, the inclusion of grey literature, while valuable for capturing policy and institutional perspectives, may involve heterogeneous methodological quality. Fourth, potential publication bias cannot be excluded, as studies reporting significant findings may be more likely to be published. Finally, the evidence base is predominantly derived from high-income countries, with limited representation of low- and middle-income settings (47, 76), which may affect the generalizability of the findings.

These limitations should be considered when interpreting the results and further highlight the need for research in more diverse and underrepresented contexts.

### 5.7 Future research

Future research should prioritize longitudinal and experimental study designs to better explore potential causal relationships between digitalization, AI, and psychosocial outcomes.

Further development and validation of measurement instruments adapted to digital work environments is also needed, as highlighted by Schlenger et al. (64) and Rodríguez (65), in order to improve the consistency and comparability of findings across studies.

Given the substantial heterogeneity identified in this review, future studies should aim to better characterize exposure to digital technologies and distinguish between different forms of digitalization and AI implementation across occupational contexts.

In addition, further research is needed to examine how organizational factors, regulatory frameworks, and digital and AI literacy influence the relationship between technological change and occupational health outcomes.

Particular attention should be paid to vulnerable populations and inequalities, including differences related to age, skill level, and access to digital resources, in order to inform more inclusive and evidence-based interventions.

Finally, future research may contribute to the development of evidence-based regulatory and organizational frameworks that support the safe, ethical, and human-centered implementation of AI in the workplace.

## 6 Conclusion

This scoping review provides a comprehensive overview of the current evidence on the impact of artificial intelligence (AI) and work digitalization on mental health, well-being, and psychosocial risks in occupational settings.

Overall, the findings indicate that digital transformation is reshaping work organization and modifying key psychosocial determinants of occupational health. Both scientific and grey literature consistently show that processes such as algorithmic management, task automation, and digital work intensification are associated with increased psychosocial risks, including technostress, work overload, reduced autonomy, and burnout, particularly when organizational resources and worker control are limited.

However, the impact of digitalization is not uniform. Evidence suggests that its effects depend largely on organizational context, job design, and the availability of resources such as training, support, and worker participation. In this regard, some studies highlight potential positive effects, showing that digital technologies may enhance efficiency, professional development, and well-being when implemented within supportive and human-centered work environments.

Institutional evidence from organizations such as the European Agency for Safety and Health at Work (EU-OSHA), the Joint Research Centre (JRC), and the International Labour Organization (ILO) reinforces these findings, emphasizing that digitalization and AI constitute emerging determinants of psychosocial risk. These bodies consistently underline the need to integrate psychosocial risk assessment into digital transformation processes and occupational health policies.

Particular attention should be paid to specific work contexts such as telework and platform-based work, which present unique psychosocial challenges, including social isolation, blurred work–life boundaries, and increased mental load. In addition, the evidence highlights that digital transformation may exacerbate structural inequalities, disproportionately affecting older workers, lower-skilled employees, and vulnerable groups.

In this context, digital and AI literacy emerge as key protective factors, contributing to greater perceived control, reduced uncertainty, and improved well-being. Strengthening these competencies, alongside promoting worker participation and maintaining human oversight in AI systems, appears essential for mitigating psychosocial risks.

Finally, this review highlights that research in this field remains in an exploratory stage. The predominance of cross-sectional studies and the limited availability of longitudinal evidence indicate the need for further research to better understand causal relationships and long-term effects. Future studies should also focus on developing evidence-based regulatory frameworks and preventive strategies that ensure a safe, equitable, and sustainable integration of AI and digital technologies in the workplace.

These findings should be interpreted within the exploratory scope of the review and in light of the heterogeneity of the available evidence.
