# Supplementary material for: Impact of artificial intelligence and work digitalization on mental health and occupational well-being: a scoping review
Source: Front Public Health. 2026 Jun 1;14:1857108. doi: 10.3389/fpubh.2026.1857108 (PMC13265473; doi:10.3389/fpubh.2026.1857108)
Supplement: Supplementary file 1 [file Supplementary_file_1.pdf]

SUPPLEMENTARY MATERIAL – APPENDIX 1

Full Search Strategies and Grey Literature Search

1.1.1 1. Overview of Search Strategy

A comprehensive literature search was conducted to identify relevant scientific evidence on the impact of artificial intelligence (AI) and work digitalization on mental health, occupational well-being, and psychosocial risks.

The search combined controlled vocabulary (e.g., MeSH terms) and free-text keywords across four conceptual domains:

- Artificial intelligence and automation
- Digitalization and technological transformation
- Occupational health and workplace context
- Mental health and psychosocial outcomes

The search covered publications from January 2016 to January 2026.
The final search was conducted on **15 January 2026**.
Language restrictions: **English and Spanish**.

Search strategies were adapted to each database to account for differences in indexing systems and syntax.

1.1.2 Electronic Database Search Strategies

PubMed (MEDLINE)

("Artificial Intelligence"[MeSH] OR "Machine Learning"[MeSH] OR "Artificial Intelligence" OR "machine learning" OR algorithm* OR automation OR "algorithmic management")
AND
("Digitization"[MeSH] OR "Digital Transformation" OR "Industry 4.0" OR digitalization OR "digital technology" OR telework OR "platform work")
AND
("Occupational Health"[MeSH] OR "Workplace" OR "Work Environment" OR "occupational safety" OR "working conditions")
AND
("Mental Health"[MeSH] OR "psychological stress" OR "psychosocial factors" OR "well-being" OR burnout OR anxiety)
Filters: 2016/01/01–2026/01/15; English, Spanish

Scopus

TITLE-ABS-KEY ("artificial intelligence" OR "machine learning" OR algorithm* OR automation OR "algorithmic management")
AND
TITLE-ABS-KEY ("digitalization" OR "digital transformation" OR "industry 4.0" OR telework OR "platform work")
AND
TITLE-ABS-KEY ("occupational health" OR workplace OR "work environment" OR "working conditions")
AND
TITLE-ABS-KEY ("mental health" OR stress OR "psychosocial risk*" OR "well-being" OR burnout)
AND PUBYEAR > 2015
AND (LIMIT-TO (LANGUAGE, "English") OR LIMIT-TO (LANGUAGE, "Spanish"))

Web of Science (Core Collection)

TS=("artificial intelligence" OR "machine learning" OR algorithm* OR automation OR "algorithmic management")
AND TS=("digitalization" OR "digital transformation" OR "industry 4.0" OR telework OR "platform work")
AND TS=("occupational health" OR workplace OR "work environment" OR "working conditions")
AND TS=("mental health" OR stress OR "psychosocial risk*" OR "well-being" OR burnout)
Refined by: Article, Review
Timespan: 2016–2026
Languages: English, Spanish

ScienceDirect

("artificial intelligence" OR "machine learning" OR algorithm OR automation)
AND
("digitalization" OR "digital transformation" OR "industry 4.0" OR telework)
AND
("occupational health" OR workplace OR "working conditions")
AND
("mental health" OR stress OR burnout OR "well-being")
Filters: 2016–2026

SciELO

("inteligencia artificial" OR "aprendizaje automático" OR algoritmo OR automatización)
AND
("digitalización" OR "transformación digital" OR teletrabajo)
AND
("salud laboral" OR trabajo OR "condiciones de trabajo")
AND
("salud mental" OR estrés OR "riesgo psicosocial" OR bienestar)
Filters: 2016–2026; Spanish, English

LILACS

("inteligencia artificial" OR "machine learning" OR algoritmo)
AND
("digitalización" OR "tecnología digital")
AND
("salud ocupacional" OR trabajo)
AND
("salud mental" OR estrés OR bienestar)
Filters: 2016–2026

Dialnet

("inteligencia artificial" OR automatización OR algoritmo)
AND
("digitalización" OR teletrabajo)
AND
("salud laboral" OR trabajo)
AND
("salud mental" OR estrés OR bienestar)
Filters: 2016–2026

Google Scholar

Searches were conducted using simplified keyword combinations:

- "artificial intelligence" AND "workplace" AND "mental health"
- "digitalization" AND "occupational health" AND stress
- "AI" AND "psychosocial risk" AND work
- First 100 results per query screened
- Sorted by relevance
- Filters: 2016–2026
